# Ultrasound Phenotype, Genetic Analysis, and Pregnancy Outcomes of Fetuses With 1p36 Deletion Syndrome

**DOI:** 10.1002/mgg3.70104

**Published:** 2025-04-26

**Authors:** Meiying Cai, Na Lin, Xuemei Chen, Hailong Huang, Nan Guo, Jiansong Lin, Liangpu Xu

**Affiliations:** ^1^ Medical Genetic Diagnosis and Therapy Center, Fujian Maternity and Child Health Hospital, College of Clinical Medicine for Obstetrics & Gynecology and Pediatrics, Fujian Medical University, Fujian Key Laboratory for Prenatal Diagnosis and Birth Defect Fuzhou China; ^2^ Department of Pathology Fujian Maternal and Child Health Hospital, College of Clinical Medicine for Obstetrics & Gynecology and Pediatrics, Fujian Medical University Fuzhou China

**Keywords:** cardiac development, prenatal, single nucleotide polymorphism array, ventriculomegaly

## Abstract

**Background:**

The intrauterine ultrasound phenotype, genotype, pregnancy outcome, and neonatal prognosis of fetuses with 1p36 deletion syndrome were retrospectively analyzed, as previous reports are limited.

**Methods:**

Pregnant women (25,000) who underwent interventional prenatal diagnosis between December 2016 and March 2024 were selected. Fetal villus tissue, amniotic fluid, or umbilical cord blood were extracted for single nucleotide polymorphism array (SNP‐array) detection under ultrasound guidance.

**Results:**

Thirteen fetuses had 1p36 deletions involving fragments that were 0.46–22.5 Mb. Six and seven fetuses had large and small copy number variation (CNV) fragment deletions in the 1p36 region, respectively. Two fetuses had normal ultrasound phenotypes, three underwent early spontaneous abortion, one had isolated ventricular septal defect, one had isolated mild ventriculomegaly, two had mild ventriculomegaly associated with increased renal echogenicity, one had mild ventriculomegaly associated with ventricular septal defect, one had severe ventriculomegaly associated with ventricular septal defect and fetal growth restriction, one had tricuspid valve dysplasia, and one had nasal bone dysplasia. Three 1p36 deletions were *de novo,* and one was paternally inherited. There were three cases of early spontaneous abortion, seven terminations, and three routine postnatal follow‐ups.

**Conclusions:**

High‐resolution SNP‐arrays are suitable for the prenatal diagnosis of 1p36 deletion syndrome.

## Graphical abstract

Phenotypic features of the 1p36 deletion syndrome in adults and children have been identified; however, due to the limitations of phenotypic identification in prenatal diagnosis, these features have not yet been described in prenatal cases. The 1p36 deletions are increasingly found by chance during prenatal testing using a single nucleotide polymorphism array (SNP‐array). There are reports on the prenatal ultrasound phenotype of the 1p36 deletion syndrome; therefore, we used an SNP‐array for genetic testing of 25,000 fetuses undergoing prenatal diagnosis at tertiary referral units.

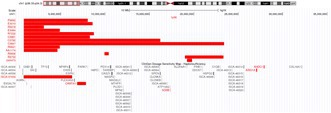

## 1 Introduction

A deletion in chromosome 1 at position 1p36, at the end of the short arm, is referred to as 1p36 deletion syndrome or 1p36 monomer syndrome. This is the most common chromosome deletion syndrome and has an incidence of approximately 0.1% in newborns and a detection rate of approximately 1% in patients with intellectual disability (Kang et al. 2016; Valerie et al. 2015). The clinical manifestations of 1p36 deletion syndrome include typical craniofacial deformities, developmental retardation, and mental retardation. In previous studies, 88% of patients with 1p36 deletion were found to have central nervous system abnormalities, 71%–75% had heart malformations, 44%–79% had seizures, 41% had skeletal abnormalities, and visual and hearing problems were also reported (Chaudhry et al. 2023; Jacquin et al. 2023; Lodato et al. 2021; Shapira et al. 1997; Zhu et al. 2013). The associated phenotypic features of 1p36 deletion syndrome in adults and children have been identified (Seeman et al. 2022). However, owing to the limitations of phenotypic identification in prenatal diagnosis, these features have not yet been systematically described in prenatal cases (Shen et al. 2024). Importantly, 1p36 deletions are increasingly identified by chance in single nucleotide polymorphism arrays (SNP‐arrays) during prenatal testing (Brisset et al. 2015; Fernandez et al. 2010).

Reports describing the prenatal ultrasound phenotypes of fetuses with 1p36 deletion syndrome are limited (Guterman et al. 2019). SNP‐arrays have a higher detection rate, resolution, and efficiency than conventional G‐banding technologies in prenatal fetal genetic disease detection. Consequently, SNP‐arrays are suitable for the prenatal diagnosis of 1p36 deletion syndrome and can provide more detailed information to aid with genetic counseling. In this study, an SNP‐array was used to analyze the genetic data of 25,000 fetuses undergoing prenatal diagnosis at tertiary referral units, of which 13 were diagnosed with 1p36 deletion syndrome. These 13 fetuses were further analyzed for intrauterine ultrasound phenotype, genotype, pregnancy outcome, and neonatal prognosis to provide evidence for the clinical management of 1p36 deletion syndrome.

## 2 Patients and Methods

### 2.1 Ethical Compliance

The studies were approved by the Ethics Committee of Fujian Provincial Maternal and Child Health Hospital. All patients consented to participate and provided signed written and informed consent. All subjects and/or their legal guardian(s) provided informed consent for study participation and the publication of identifying information/images in an online open‐access publication. All methods were performed in accordance with the relevant guidelines and regulations.

### 2.2 Patient Recruitment

This retrospective study included 25,000 pregnant women who underwent SNP‐array analysis for interventional prenatal diagnosis at tertiary referral centers between December 2016 and March 2024. Villus tissue was extracted based on the gestational age using ultrasound‐guided choriocentesis (11–14 weeks of gestation), amniotic fluid extraction using amniocentesis (16–24 weeks of gestation), or cord blood extraction using umbilical vein puncture (> 24 weeks of gestation) for SNP‐array detection. The pregnant women were 28 ± 5 years of age, and the gestational age was 23 ± 1 weeks. This study was approved by the Ethics Committee of Fujian Maternal and Child Health Hospital (2014042), and all parents of the included fetuses provided signed informed consent.

### 2.3 Single Nucleotide Polymorphism Array

Fetal genomic DNA was extracted using a QIAamp DNA Blood Mini Kit (Qiagen, Germany). A CytoScan750k Chip Kit (Affymetrix, USA) was used to cross the sample DNA with a gene chip containing a high density of SNP and copy number variation (CNV) probes for detection at the whole‐genome level. The basic principles for classifying CNV results into three broad categories and five levels were in accordance with the American Society for Medical Genetics and Genomics guidelines (Riggs et al. 2021), and the categories were as follows: (1) pathogenic CNV, (2) likely pathogenic CNV, (3) variant of uncertain significance (VUS), (4) likely benign CNV, and (5) benign CNV. Peripheral blood samples from the parents of VUS fetuses were analyzed using SNP‐arrays, and the final nature of the CNV was determined in combination with the family line. International public genetic databases, including the Database of Genomic Variants, Database of Chromosomal Imbalance and Phenotype in Humans Using Ensemble Resources, Database of Chromosomal Imbalance and Phenotype in humans using Ensemble Resources, and online database resources, such as DECIPHER, Clinical Genome Resource, and Online Mendelian Inheritance in Man, were used to determine the dose sensitivity of CNVs and elucidate the correlations between CNV, clinical phenotype, and prognosis. The reference genome version is GRCh37 (hg19).

### 2.4 Pregnancy Outcome Follow‐Up

Clinical data were obtained from prenatal diagnoses, and follow‐up records of pregnancy outcomes and individual growth and development were monitored.

## 3 Results

### 3.1 SNP‐Array Results

Of the 25,000 fetuses tested using the SNP‐array, 13 were found to have deletions in the 1p36 region, with a detection rate of 0.5% (13/25,000). Of these, six had large CNV fragment deletions and seven had CNV microdeletions in the 1p36 region.

### 3.2 Molecular Characterization and Ultrasound Phenotype of Fetuses With 1p36 Deletion Syndrome

**FIGURE 1:**
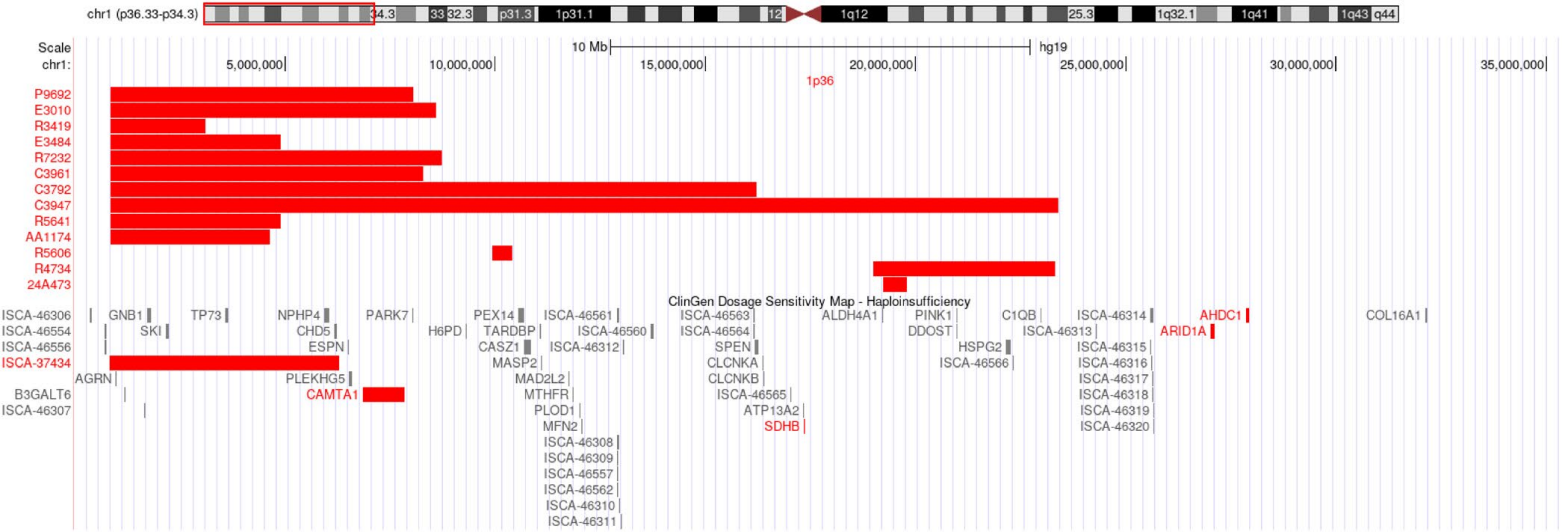
SNP‐array data for 13 fetuses with deletions in the 1p36 region. The reference genome version is GRCh37 (hg19).

Thirteen fetuses had CNV deletions in the 1p36 region, which involved fragments from 0.46 to 22.5 Mb and contained genes such as *GABRD* (OMIM:137163) (NG 008168.1), *CAMTA1* (OMIM:614756) (NG 053148.1), and *GNB1* (OMIM:139380) (NG 047052.1) (Figure 1). The indications for invasive genetic testing in the 13 fetuses with 1p36 deletions were noninvasive prenatal testing positive (two cases), early spontaneous abortion (three cases), and ultrasound abnormality (eight cases). Among the eight fetuses with ultrasound abnormalities, one had isolated ventricular septal defect, one had isolated mild ventriculomegaly, two had mild ventriculomegaly associated with increased renal echogenicity, one had mild ventriculomegaly associated with ventricular septal defect, and one had severe ventriculomegaly associated with ventricular septal defect and fetal growth restriction. There was also one case of tricuspid valve dysplasia and one case of nasal bone dysplasia (Table 1). After genetic counseling, the parents of eight fetuses with 1p36 deletion syndrome refused family verification. The parents of five fetuses with 1p36 deletion syndrome allowed for family verification, and the results showed that four cases were *de novo*, while one was a result of paternal inheritance.

**TABLE 1:** Clinical features of 13 fetuses with the 1p36 deletion syndrome.

Case	Prenatal diagnostic pointer	SNP‐array	Inclusion gene	Size (Mb)	Inheritance	Pregnancy outcome
R7232	NIPT positive	arr[hg19]1p36.33p36.23(849,467–8,731,413)x1	*GABRD, CAMTA1*	7.8	—	TP
P9692	Ventricular septal defect	arr[hg19]1p36.33p36.23(849,466–8,056,475)x1	*GABRD, CAMTA1*	7.2	—	TP
E3010	Mild ventriculomegaly	arr[hg19]1p36.33p36.23(849,466–8,592,172)x1	*GABRD, CAMTA1*	7.7	—	TP
R4734	Nasal bone dysplasia	arr[hg19]1p36.13p36.12(18,987,622–23,321,266)x1	*CDC42, EPHB2*	4.3	*De novo*	TP
C3961	Early spontaneous abortion	arr[hg19]1p36.33p36.23(849,467–8,284,657)x1	*GABRD, GNB1*	7.4	—	Early spontaneous abortion
C3792	Early spontaneous abortion	arr[hg19]1p36.33p36.13(849,467–16,217,411)x1	*CAMTA1*	15.3	—	Early spontaneous abortion
C3947	Early spontaneous abortion	arr[hg19]1p36.33p36.12(849,467–23,387,403)x1	*GABRD, CAMTA1*	22.5	—	Early spontaneous abortion
R5641	Mild ventriculomegaly, increased renal echogenicity	arr[hg19]1p36.33p36.32(849,467–4,894,800)x1	*GABRD, GNB1*	4.0	*De novo*	1 year 2 months, healthy
R3419	Mild ventriculomegaly, ventricular septal defect	arr[hg19]1p36.33p36.32(849,466–3,101,616)x1	*GABRD, GNB1*	2.2	*De novo*	TP
E3484	Mild ventriculomegaly, renal echo enhancement	arr[hg19]1p36.33p36.32(849,466–4,894,800)x1,11p15.5p15.4(230,680–8,918,951)x3	*GABRD, GNB1*	4.0	—	TP
AA1174	Severe ventriculomegaly, ventricular septal defect, fetal growth restriction	arr[hg19]1p36.33p36.32(849,467–4,629,193)x1	*GABRD, GNB1*	3.78	—	TP
24A473	NIPT positive	arr[hg19]1p36.13(19,223,454–19,796,787)x1	*ALDH4A1*	0.56	*De novo*	Six months, healthy
R5606	Tricuspid valve dysplasia, separation of the renal collecting system	arr[hg19]1p36.22(9,925,708–10,398,985)x1	*NMNAT1, KIF1B*	0.46	Pat	2 years old, healthy

*Note:* The reference genome version is GRCh37 (hg19).

Abbreviations: NIPT, noninvasive prenatal testing; TP, termination of pregnancy.

### 3.3 Pregnancy Outcomes

Of the 13 cases with 1p36 deletion syndrome, three were miscarried early and the other 10 were informed of the possible risks after adequate genetic counseling. The parents of seven of the fetuses chose to terminate the pregnancy, and the parents of the remaining three chose to continue the pregnancy (Table 1). No abnormalities were found in the three fetuses with one p36 deletion syndrome after delivery. At the time of writing, the youngest infant was 6 months old and the oldest was 2 years old.

## 4 Discussion

Although the incidence of 1p36 deletion syndrome is high, only a few cases have been clinically confirmed, primarily because the clinical phenotypic characteristics are not yet well‐established and traditional detection methods are limited (Zenker et al. 2002). Most patients have been diagnosed using cytogenetic methods; however, abnormalities in small segments around 1p36 are difficult to detect because of the large number of genes in the short arm subtelomere region of chromosome 1 and the low resolution of chromosomal karyotyping (Esplin 2020). In this study, 13 fetuses with 1p36 deletions were identified, among which six had large genome copy number deletion fragments in the 1p36 region, which could be detected using karyotyping. The remaining seven fetuses had microdeletions in the 1p36 region that karyotyping could not detect. SNP‐arrays have been widely used for prenatal diagnosis because of their high resolution and ability to detect deletions and duplications > 5 Mb, which can also be detected by karyotyping. SNP‐arrays are especially suitable for detecting fragment deletions and duplications < 50 kb, providing an incomparable advantage over traditional karyotype analysis (Wou et al. 2016). SNP‐arrays are the most effective method currently available for detecting 1p36 deletion syndrome. The data from this study provide a basis for detecting, treating, and preventing abnormal fetuses (Bejjani and Shaffer 2006).

Four chromosomal rearrangements were observed in patients with 1p36 deletion syndrome: simple terminal deletion (67%), derived chromosome deletion (16%), intermediate deletion (10%), and complex rearrangement (7%) (Gajecka et al. 2007). The different sizes and locations of the missing fragments in patients with 1p36 deletion syndrome resulted in different haplodose–underdose effects because of different dose‐sensitive gene deletions, different gene imprinting effects, and exposure to specific recessive mutation alleles, resulting in a diverse array of phenotypes (Welham et al. 2015). The 1p36 region contains many genes, and some candidate genes related to clinical phenotypes have been identified. Deletion of *GABRD* can lead to insufficient gene expression haplodoses, causing changes in the ion channels that can lead to epileptic symptoms (Ahring et al. 2022). *GNB1* encodes a subunit of the heterotrimer G‐protein complex that transduces intracellular signaling cascades. Deletion of *GNB1* can lead to associated neurodevelopmental disorders (Colombo et al. 2023). Loss of *CDC42* (OMIM:116952) (NG 000001.1) reduces the expression level of protein 2 (dHand) expressed by cardiac and neural crest derivatives, and this affects right ventricular development, which can lead to cardiac developmental defects (Garcia‐Padilla et al. 2022; Liu et al. 2017). The brain‐specific transcription factor *CAMTA1* binds to the CGCG box of gene promoters via the CG‐1 domain, supporting the assembly of other transcription factors and enhancing effector gene transcription. When the haplodose of *CAMTA1* is insufficient, the deletion or duplication of the gene region encoding the CG‐1 domain will affect the function of *CAMTA1*, resulting in cerebellar abnormalities, ventricle enlargement, with or without related hydrocephalus, and developmental delay (Wijnen et al. 2020). Clinical phenotypes, such as mental retardation and epilepsy, cannot be detected using ultrasonography before birth. Despite the high incidence of 1p36 deletions, prenatal diagnoses are rare. In the reported prenatal diagnoses, the ultrasound phenotypes of fetuses with 1p36 deletion syndrome predominantly included ventriculomegaly, abnormal brain development, and cardiac abnormalities (Campeau et al. 2010; Cavani et al. 2010; Guterman et al. 2019; Song et al. 2024). During brain development in fetuses with a 1p36 deletion of the abnormalities were predominantly in corpus callosum development and cerebellar vermis deletion (Guterman et al. 2019). Cardiac abnormalities in LP3‐absent fetuses include ventricular septal defects, persistent trunk arteries, and tricuspid valve agenesis (Lodato et al. 2021). The ultrasonic phenotypes of 1p36 deletion fetuses can also include intrauterine growth retardation, hydronephrosis, and a single umbilical artery (Zhang et al. 2021). Among the 13 fetuses with 1p36 deletion syndrome reported in this study, one had an isolated ventricular septal defect, one had an isolated mild ventriculomegaly, two had mild ventriculomegaly associated with increased renal echogenicity, one had mild ventriculomegaly associated with ventricular septal defect, and one had severe ventriculomegaly associated with ventricular septal defect and fetal growth restriction. Thus, identifying ventriculomegaly or cardiac abnormalities in prenatal ultrasounds could be used to identify 1p36 deletion syndrome.

The occurrences of 1p36 deletion syndrome are usually sporadic (Gajecka et al. 2015). In this study, of the 13 fetuses identified with the 1p36 deletion syndrome, family verification of the parents of five fetuses showed that four cases had *de novo* inheritance. In contrast, the fifth case showed paternal inheritance. The phenotypes of lp36 deletion syndrome patients have demonstrated high levels of heterogeneity. When a fetus with lp36 deletion syndrome has a severe intrauterine phenotype, the pregnant woman should be advised to terminate the pregnancy immediately. When the fetus with lp36 deletion syndrome has a normal intrauterine ultrasound phenotype, which is inherited by the parents, and the parent carrier has a normal phenotype, the prognosis of this fetus is better. Therefore, in prenatal genetic counseling, pregnant women and their families should be assisted to make the right choice under fully informed conditions. After genetic counseling to inform the parents of 10 fetuses with 1p36 deletion syndrome of the possible risks, the parents of seven fetuses chose to terminate their pregnancies. In comparison, three fetuses with 1p36 deletions were delivered normally, and no abnormalities were identified during their follow‐ups. The intrauterine ultrasound phenotype of a fetus with a *de novo* 4 Mb fragment size 1p36 deletion showed ventriculomegaly and enhanced renal echo. Follow‐ups for 1 year and 2 months after birth revealed no abnormal clinical phenotypes. One fetus with a *de novo* 0.56 Mb fragment size 1p36 deletion had a normal intrauterine ultrasound phenotype, and follow‐ups for 6 months after birth did not report any abnormal clinical phenotype. The intrauterine ultrasound phenotype of a fetus with a 0.56 Mb fragment size 1p36 deletion inherited from the father showed tricuspid valve dysplasia. Follow‐ups for 2 years after birth revealed no abnormal clinical phenotypes. Overall, the three live births with 1p36 deletions reported in this study showed no abnormal clinical phenotypes after birth. This may be due to the small fragment size of the 1p36 deletion area or the short follow‐up times, as an abnormal clinical phenotype may not have had sufficient time to manifest. In the future, we will continue to follow up and track the three cases with 1p36 deletions and collect additional case data to help improve our understanding of the ultrasound phenotypes of fetuses with 1p36 deletion syndrome and thus better guide clinical prenatal work.

This study has some limitations. First, whole exome sequencing was not applied (Alamillo et al. 2015; Carss et al. 2014; Chandler et al. 2018). Whole‐exome sequencing can identify genetic variations or hereditary syndromes that cause fetal abnormalities. These data could improve the clinical management of hereditary syndromes and are more comprehensive than SNP‐array data. Second, the identified live births with the 1p36 deletion syndrome were only tracked for 2 years after birth. Clinical phenotypes, such as intellectual disability and epilepsy, generally only manifest in slightly older children. To improve clinical interventions, long‐term follow‐ups of these cases will be necessary in the future. Third, the sample size of this study was relatively small. In future studies, we will incorporate larger cohorts or multicenter data collection.

In conclusion, SNP‐arrays are advantageous because they are high‐resolution and thus suitable for the prenatal diagnosis of 1p36 deletion syndrome, providing more detailed information for genetic counseling. Prenatal ultrasound revealed ventriculomegaly and abnormal cardiac development, indicating the possibility of 1p36 deletion syndrome.

## Author Contributions

Liangpu Xu and Na Lin designed and supervised the project. Meiying Cai and Hailong Huang wrote and revised the manuscript. Xuemei Chen provided clinical data. Nan Guo and Meimei Fu performed data analysis and variant interpretation. All authors read and approved the final manuscript.

## Ethics Statement

The studies were approved by the Ethics Committee of Fujian Provincial Maternal and Child Health Hospital. All patients consented to participate and provided signed written and informed consent. All subjects and/or their legal guardian(s) provided informed consent for study participation and the publication of identifying information/images in an online open‐access publication. All methods were performed in accordance with the relevant guidelines and regulations.

## Consent

Written informed consent was obtained from the participants for publication of this article and any accompanying tables/images.

## Conflicts of Interest

The authors declare no conflicts of interest.

## Data Availability

The data supporting this study's findings are available from the corresponding author upon reasonable request.

## References

[mgg370104-bib-0001] Ahring, P. K. , V. W. Y. Liao , E. Gardella , et al. 2022. “Gain‐of‐Function Variants in GABRD Reveal a Novel Pathway for Neurodevelopmental Disorders and Epilepsy.” Brain 145, no. 4: 1299–1309. 10.1093/brain/awab391. 34633442 PMC9630717

[mgg370104-bib-0002] Alamillo, C. L. , Z. Powis , K. Farwell , et al. 2015. “Exome Sequencing Positively Identified Relevant Alterations in More Than Half of Cases With an Indication of Prenatal Ultrasound Anomalies.” Prenatal Diagnosis 35, no. 11: 1073–1078. 10.1002/pd.4648. 26147564

[mgg370104-bib-0003] Bejjani, B. A. , and L. G. Shaffer . 2006. “Application of Array‐Based Comparative Genomic Hybridization to Clinical Diagnostics.” Journal of Molecular Diagnostics 8, no. 5: 528–533. 10.2353/jmoldx.2006.060029. PMC1876176 17065418

[mgg370104-bib-0004] Brisset, S. , Y. Capri , A. Briand‐Suleau , et al. 2015. “Inherited 1q21.1q21.2 Duplication and 16p11.2 Deletion: A Two‐Hit Case With More Severe Clinical Manifestations.” European Journal of Medical Genetics 58, no. 9: 497–501. 10.1016/j.ejmg.2015.07.001. 26162704

[mgg370104-bib-0005] Campeau, P. M. , N. A. Mew , L. Cartier , et al. 2010. “Prenatal Diagnosis of Monosomy 1p36: A Focus on Brain Abnormalities and a Review of the Literature.” American Journal of Medical Genetics Part A 146A, no. 23: 3062–3069. 10.1002/ajmg.a.32563 19006213

[mgg370104-bib-0006] Carss, K. J. , S. C. Hillman , V. Parthiban , et al. 2014. “Exome Sequencing Improves Genetic Diagnosis of Structural Fetal Abnormalities Revealed by Ultrasound.” Human Molecular Genetics 23, no. 12: 3269–3277. 10.1093/hmg/ddu038. 24476948 PMC4030780

[mgg370104-bib-0007] Cavani, S. , C. Perfumo , F. Faravelli , et al. 2010. “Cryptic 1p36.3/6q25.2 Translocation in Three Generations Ascertained Through a Foetus With IUGR and Cerebral Malformations.” Prenatal Diagnosis 23, no. 10: 819–823. 10.1002/pd.678 14558026

[mgg370104-bib-0008] Chandler, N. , S. Best , J. Hayward , et al. 2018. “Rapid Prenatal Diagnosis Using Targeted Exome Sequencing: A Cohort Study to Assess Feasibility and Potential Impact on Prenatal Counseling and Pregnancy Management.” Genetics in Medicine 20, no. 11: 1430–1437. 10.1038/gim.2018.30. 29595812

[mgg370104-bib-0009] Chaudhry, C. , D. Kumari , I. Panigrahi , and P. Kaur . 2023. “Chromosome 1p36 Deletion Syndrome: Four Patients With Variable Presentations.” Journal of Pediatric Genetics 12, no. 4: 342–347. 10.1055/s-0041-1732477. 38162157 PMC10756721

[mgg370104-bib-0010] Colombo, S. , H. P. Reddy , S. Petri , et al. 2023. “Epilepsy in a Mouse Model of GNB1 Encephalopathy Arises From Altered Potassium (GIRK) Channel Signaling and Is Alleviated by a GIRK Inhibitor.” Frontiers in Cellular Neuroscience 17: 1175895. 10.3389/fncel.2023.1175895. 37275776 PMC10232839

[mgg370104-bib-0011] Esplin, E. D. 2020. “Addendum: American College of Medical Genetics Guideline on the Cytogenetic Evaluation of the Individual With Developmental Delay or Mental Retardation.” Genetics in Medicine 22, no. 12: 2128. 10.1038/s41436-020-0875-5. 32624573

[mgg370104-bib-0012] Fernandez, B. A. , W. Roberts , B. Chung , et al. 2010. “Phenotypic Spectrum Associated With De Novo and Inherited Deletions and Duplications at 16p11.2 in Individuals Ascertained for Diagnosis of Autism Spectrum Disorder.” Journal of Medical Genetics 47, no. 3: 195–203. 10.1136/jmg.2009.069369. 19755429

[mgg370104-bib-0013] Gajecka, M. , K. L. Mackay , and L. G. Shaffer . 2007. “Monosomy 1p36 Deletion Syndrome.” American Journal of Medical Genetics. Part C, Seminars in Medical Genetics 145C, no. 4: 346–356. 10.1002/ajmg.c.30154. 17918734

[mgg370104-bib-0014] Gajecka, M. , S. C. Saitta , A. J. Gentles , L. Campbell , and L. G. Shaffer . 2015. “Recurrent Interstitial 1p36 Deletions: Evidence for Germline Mosaicism and Complex Rearrangement Breakpoints.” American Journal of Medical Genetics Part A 152A, no. 12: 3074–3083. 10.1002/ajmg.a.33733 PMC3058890 21108392

[mgg370104-bib-0015] Garcia‐Padilla, C. , V. Garcia‐Lopez , A. Aranega , D. Franco , V. Garcia‐Martinez , and C. Lopez‐Sanchez . 2022. “Inhibition of RhoA and Cdc42 by miR‐133a Modulates Retinoic Acid Signaling During Early Development of Posterior Cardiac Tube Segment.” International Journal of Molecular Sciences 23, no. 8: 4179. 10.3390/ijms23084179. 35456995 PMC9025022

[mgg370104-bib-0016] Guterman, S. , C. Beneteau , S. Redon , et al. 2019. “Prenatal Findings in 1p36 Deletion Syndrome: New Cases and a Literature Review.” Prenatal Diagnosis 39, no. 10: 871–882. 10.1002/pd.5498. 31172545

[mgg370104-bib-0017] Jacquin, C. , E. Landais , C. Poirsier , et al. 2023. “1p36 Deletion Syndrome: Review and Mapping With Further Characterization of the Phenotype, a New Cohort of 86 Patients.” American Journal of Medical Genetics. Part A 191, no. 2: 445–458. 10.1002/ajmg.a.63041. 36369750 PMC10100125

[mgg370104-bib-0018] Kang, D. S. , E. Shin , and J. Yu . 2016. “1p36 Deletion Syndrome Confirmed by Fluorescence In Situ Hybridization and Array‐Comparative Genomic Hybridization Analysis.” Korean Journal of Pediatrics 59, no. Suppl 1: S14–S18. 10.3345/kjp.2016.59.11.S14. 28018437 PMC5177698

[mgg370104-bib-0019] Liu, Y. , J. Wang , J. Li , et al. 2017. “Deletion of Cdc42 in Embryonic Cardiomyocytes Results in Right Ventricle Hypoplasia.” Clinical and Translational Medicine 6, no. 1: 40. 10.1186/s40169-017-0171-4. 29101495 PMC5670094

[mgg370104-bib-0020] Lodato, V. , V. Orlando , V. Alesi , et al. 2021. “1p36 Deletion Syndrome and the Aorta: A Report of Three New Patients and a Literature Review.” Journal of Cardiovascular Development and Disease 8, no. 11: 159. 10.3390/jcdd8110159. 34821712 PMC8618808

[mgg370104-bib-0021] Riggs, E. R. , E. F. Andersen , A. M. Cherry , et al. 2021. “Correction: Technical Standards for the Interpretation and Reporting of Constitutional Copy‐Number Variants: A Joint Consensus Recommendation of the American College of Medical Genetics and Genomics (ACMG) and the Clinical Genome Resource (ClinGen).” Genetics in Medicine 23, no. 11: 2230. 10.1038/s41436-021-01150-9. 33731880

[mgg370104-bib-0022] Seeman, P. , V. Cejnova , S. Cerna , et al. 2022. “Czech Family Confirms the New 1p36.13‐1p36.12 Microdeletion Syndrome.” Clinical Genetics 102, no. 3: 244–245. 10.1111/cge.14175. 35726688

[mgg370104-bib-0023] Shapira, S. K. , C. Mccaskill , H. Northrup , et al. 1997. “Chromosome 1p36 Deletions: The Clinical Phenotype and Molecular Characterization of a Common Newly Delineated Syndrome.” American Journal of Human Genetics 61, no. 3: 642–650. 9326330 10.1086/515520 PMC1715949

[mgg370104-bib-0024] Shen, Y. , W. Zhang , P. Hua , and F. Qian . 2024. “Genetic Analysis of 1p36 Deletions for Six Aborted Fetuses.” Alternative Therapies in Health and Medicine 61: 10. 38401069

[mgg370104-bib-0025] Song, T. , J. Zheng , Y. Li , et al. 2024. “Prenatal Diagnosis of Pure 1p36 Terminal Deletion by Chromosome Microarry Analysis—Clinical Report of 3 New Cases and Review of the Literature.” Ginekologia Polska 95, no. 12: 947–951. 10.5603/GP.a2021.0173. 35072240

[mgg370104-bib-0026] Valerie, J. , H. Zaveri , and J. Scott . 2015. “1p36 Deletion Syndrome: An Update.” Application of Clinical Genetics 5: 189–200. 10.2147/TACG.S65698 PMC4555966 26345236

[mgg370104-bib-0027] Welham, A. , J. Lau , J. Moss , et al. 2015. “Are Angelman and Prader‐Willi Syndromes More Similar Than We Thought? Food‐Related Behavior Problems in Angelman, Cornelia de Lange, Fragile X, Prader‐Willi and 1p36 Deletion Syndromes.” American Journal of Medical Genetics Part A 167, no. 3: 572–578. 10.1002/ajmg.a.36923 25691410

[mgg370104-bib-0028] Wijnen, I. G. M. , H. E. Veenstra‐Knol , F. Vansenne , et al. 2020. “De Novo Variants in CAMTA1 Cause a Syndrome Variably Associated With Spasticity, Ataxia, and Intellectual Disability.” European Journal of Human Genetics 28, no. 6: 763–769. 10.1038/s41431-020-0600-5. 32157189 PMC7253440

[mgg370104-bib-0029] Wou, K. , B. Levy , and R. J. J. C. Wapner . 2016. “Chromosomal Microarrays for the Prenatal Detection of Microdeletions and Microduplications.” Clinics in Laboratory Medicine 36, no. 2: 261–276. 27235911 10.1016/j.cll.2016.01.017

[mgg370104-bib-0030] Zenker, M. , O. Rittinger , K. P. Grosse , et al. 2002. “Monosomy 1p36—a Recently Delineated, Clinically Recognizable Syndrome.” Clinical Dysmorphology 11, no. 1: 43–48. 10.1097/00019605-200201000-00009. 11822705

[mgg370104-bib-0031] Zhang, X. , P. He , J. Han , et al. 2021. “Prenatal Detection of 1p36 Deletion Syndrome: Ultrasound Findings and Microarray Testing Results.” Journal of Maternal‐Fetal & Neonatal Medicine 34, no. 13: 2180–2184. 10.1080/14767058.2019.1660764. 31446820

[mgg370104-bib-0032] Zhu, X. , Y. Zhang , J. Wang , J. F. Yang , Y. F. Yang , and Z. P. Tan . 2013. “576kb Deletion in 1p36.33–p36.32 Containing SKI Is Associated With Limb Malformation, Congenital Heart Disease and Epilepsy.” Gene 528, no. 2: 352–355. 23892090 10.1016/j.gene.2013.07.024

